# Hippocampal-Cortical Memory Trace Transfer and Reactivation Through Cell-Specific Stimulus and Spontaneous Background Noise

**DOI:** 10.3389/fncom.2019.00067

**Published:** 2019-09-24

**Authors:** Xin Liu, Duygu Kuzum

**Affiliations:** Department of Electrical and Computer Engineering, University of California, San Diego, San Diego, CA, United States

**Keywords:** memory transfer, hippocampus model, sequence replay, prefrontal cortex model, hippocampal-cortical network

## Abstract

The hippocampus plays important roles in memory formation and retrieval through sharp-wave-ripples. Recent studies have shown that certain neuron populations in the prefrontal cortex (PFC) exhibit coordinated reactivations during awake ripple events. These experimental findings suggest that the awake ripple is an important biomarker, through which the hippocampus interacts with the neocortex to assist memory formation and retrieval. However, the computational mechanisms of this ripple based hippocampal-cortical coordination are still not clear due to the lack of unified models that include both the hippocampal and cortical networks. In this work, using a coupled biophysical model of both CA1 and PFC, we investigate possible mechanisms of hippocampal-cortical memory trace transfer and the conditions that assist reactivation of the transferred memory traces in the PFC. To validate our model, we first show that the local field potentials generated in the hippocampus and PFC exhibit ripple range activities that are consistent with the recent experimental studies. Then we demonstrate that during ripples, sequence replays can successfully transfer the information stored in the hippocampus to the PFC recurrent networks. We investigate possible mechanisms of memory retrieval in PFC networks. Our results suggest that the stored memory traces in the PFC network can be retrieved through two different mechanisms, namely the cell-specific input representing external stimuli and non-specific spontaneous background noise representing spontaneous memory recall events. Importantly, in both cases, the memory reactivation quality is robust to network connection loss. Finally, we find out that the quality of sequence reactivations is enhanced by both increased number of SWRs and an optimal background noise intensity, which tunes the excitability of neurons to a proper level. Our study presents a mechanistic explanation for the memory trace transfer from the hippocampus to neocortex through ripple coupling in awake states and reports two different mechanisms by which the stored memory traces can be successfully retrieved.

## Introduction

The hippocampus (HPC) plays important roles in memory consolidation and sharp-wave ripples (SWR) are believed to transfer the compressed temporary information stored in the hippocampus to the distributed cortical networks (Buzsáki, 1989; Diekelmann and Born, 2010) through the abundant connections between the hippocampus and the cortex. Systems consolidation theory hypothesizes that memory consolidation process redistribute the hippocampal-dependent memories to support integration of the newly acquired memories with the related existing ones by reorganizing the cortical networks (Squire and Alvarez, 1995; Dudai et al., 2015). In support of this idea, it has been shown that during sleep, the neurons in prefrontal cortex (PFC) display learning-dependent reactivations when SWR are generated (Peyrache et al., 2009). Furthermore, the firing of PFC neurons falls within the plasticity time window after HPC SWR occurs (Wierzynski et al., 2009). It has also been reported that enhancing the oscillation coupling between HPC and PFC boosts the memory task performance (Maingret et al., 2016). Importantly, during sleep the neurons in PFC can also exhibit fast sequential reactivations with a compression factor of 6–7 compared to that during behavioral states (Euston et al., 2007). These experimental findings support the view that HPC SWR indeed play important roles in memory consolidation during sleep.

Besides sleep, SWR and sequence replay also occur in the HPC during awake immobility (Foster and Wilson, 2006; Diba and Buzsáki, 2007; Karlsson and Frank, 2009). Similar with sleep SWR, interruption of awake SWR during spatial learning degrades the animal’s performance in later spatial tasks (Jadhav et al., 2012). Recent studies have demonstrated strong hippocampal-cortical modulations during awake SWR. The PFC neurons reactivate during awake SWR and different excitation and inhibition patterns have been observed in the PFC neuron populations (Jadhav et al., 2016). Also, the CA1-PFC reactivation has been found to be stronger during awake SWR than during sleep SWR. Especially when the animal is learning novel information, the CA1-PFC reactivation is further enhanced (Tang et al., 2017). These observations lead to the postulation that HPC not only contributes to memory consolidation during sleep, but also takes active part in the hippocampus-dependent memory retrieval and initial learning process in the cortex during awake behavior. Previously, computational modeling work in literature have focused on modeling the generation of SWRs (Cutsuridis and Taxidis, 2013; Fink et al., 2015; Taxidis et al., 2015; Malerba and Bazhenov, 2018) and theta oscillations in hippocampal networks (Cutsuridis and Hasselmo, 2012; Bezaire et al., 2016), as well as the mechanism of memory encoding and retrieval in CA1 network (Cutsuridis et al., 2010; Cutsuridis, 2019) and CA3 network (Kunec et al., 2005; Saravanan et al., 2015). Other computational models have also been built to study the mechanisms of neural activities observed in PFC networks (Durstewitz and Gabriel, 2006; Hass et al., 2016). A recent study has investigated the modulation of slow oscillations in PFC network on the hippocampal activity in CA3 and CA1 networks (Taxidis et al., 2013). A unified model that connects and couples the hippocampal and prefrontal networks is especially important to investigate the mechanisms by which the HPC SWRs assist memory consolidation and retrieval.

Toward this goal, we build a unified biophysical computational model that couples a hippocampal CA1 network (Canakci et al., 2017) with a PFC network to study the memory transfer from the HPC to the PFC and memory trace reactivations. Under the sequential inputs from a virtual CA3 network, the CA1 network generates ripple range oscillations in the local field potentials along with simultaneous sequential pyramidal cell replays. The firing activity in CA1 potentiates the pyramidal cells in PFC through the monosynaptic connections to induce coordinated sequence reactivations. We demonstrate that the sequence can be transferred and stored in the recurrent connections of the PFC network through spike-timing-dependent plasticity (STDP) between PFC neurons. Later, we find that the stored memory traces can be reactivated through two different mechanisms, namely the cell-specific local stimulation and non-specific spontaneous background noise. Interestingly, in both conditions, the memory trace reactivation is robust to the connections loss between the pyramidal cells in the PFC network. Finally, the reactivation quality in the PFC network can be improved by either by increased number of SWRs or adjusting the background noise level to optimal value. Our work provides a mechanistic model for the hippocampus dependent memory formation through SWR based cortex–hippocampus interactions in awake state and examines neuronal and network level parameters affecting spontaneous and stimulus-induced memory retrieval.

## Materials and Methods

### Hippocampus Model

The hippocampus CA1 model is based on the previous work, which has demonstrated the generation of sharp-wave-ripples under noisy inputs (Stacey et al., 2009; Fink et al., 2015; Canakci et al., 2017). Our adapted model consists of 400 pyramidal cells (PY cells) and 100 basket cells (BS cells), which are chosen to comply with the CA1 neuroanatomy (Andersen et al., 2006; Bezaire and Soltesz, 2013) and other CA1 modeling work (Tort et al., 2007; Stacey et al., 2009; Malerba and Bazhenov, 2018). Note that even if the ratio may not be exact compared to the real biological CA1 network, the net inhibition or excitation can always be compensated by adjusting the relative synaptic strength between pyramidal and interneurons to accurately model SPW-Rs. Similar to the previous publications (Tort et al., 2007; Stacey et al., 2009; Fink et al., 2015), the pyramidal cell model has five compartments, namely the soma, the basal dendrite, and three apical dendrites. Since the axon has small surface area and does not contribute much to the LFP recordings, we do not explicitly model it. The basket cell is modeled as a three-compartment soma, without dendrite and axons. The geometry of each compartment is listed in Supplementary Table S1.

The connections between different neurons in CA1 network are listed in Supplementary Table S2. Based on the anatomical study of the connectivity in CA1 area (Bezaire and Soltesz, 2013), each BS cell receives excitatory AMPA projections from the nearest 50 PY cells, while each PY cell receives inhibitory GABA projections from the nearest 20 BS cells. Since gap junctions were identified in a small area within 200 μm between basket cells in previous experimental studies (Fukuda and Kosaka, 2000; Tamás et al., 2000; Bartos et al., 2001), each basket cell in our model forms gap junctions with the nearest 4 BS cells. The gap junction between BS cells has been incorporated in previous modeling studies and is believed to contribute to the synchrony of hippocampal ripples (Saraga et al., 2006; Holzbecher and Kempter, 2018). The specifics of the synapse location, strength, and delay are also summarized in Supplementary Table S2.

In our CA1 network model, 400 PY cells are randomly allocated into five different groups, each of which has 80 PY cells and represents one place cell assembly, as observed in experimental studies (Holtmaat and Caroni, 2016). To mimic the synaptic input from CA3, each place cell assembly receives sequential Poisson noisy input. The parameters of the input is summarized in Supplementary Table S3. The setting of this CA3 input pattern is based on recent experimental studies, which show that during awake SWR associated replay, the decoded trajectory from the firing activities of CA1 place cells exhibit discrete “jump” behaviors, which is believed to result from the attractor state changes in the upstream CA3 (Pfeiffer and Foster, 2015). Since the reported mean time for the decoded location change during one ripple event falls within the 25–50 Hz slow gamma range, we choose to set the duration of each noisy input to be consistent within this range.

### Prefrontal Cortex Model

Our model for PFC is based on the layer V PFC microcircuit model published in Papoutsi et al. (2013, 2014). We modified the original model to include 100 pyramidal (PY) cells and 25 interneurons (IN). The biophysical properties of the single neuron model are kept the same. Similar to the CA1 network model, the PY cells and IN cells in PFC model also form two 2D planes with 10 um spacing and 20 um spacing, respectively. The physical specifics of the cell model is shown in Supplementary Table S4. To reduce the computational complexity of the network and to be consistent with our CA1 model, we reduce the segment number of each compartment to 1.

The connections between the cells in the PFC network is shown in Supplementary Table S5. It has been reported that one PY cell in rat visual cortex makes unidirectional and bidirectional connections to other PY cells with a probability of 0.13 and 0.06 (Song et al., 2005). Since the PFC area has abundant recurrent PY-PY connections, which are more than double the rate than in visual cortex (Wang et al., 2006), we set the probability of unidirectional connection to 0.25 and the probability of bidirectional connection to 0.12. For the IN cells, each of them is connected to randomly chosen 10 IN cells based on previous anatomical studies (Pfeffer et al., 2013).

During wakefulness, the membrane potentials of the cortical neurons undergo persistent depolarization, which is quite different from that during asleep or anesthetized states (Constantinople and Bruno, 2011). To simulate the background noise on the PY cells and IN cells in PFC, we add independent Poisson inputs to the dendrites of PY cells and soma of IN cells. As a result, the PY cells and IN cells exhibit stochastic firing activities with frequencies of 0.11 and 9.25 Hz, respectively, which are consistent with the reported PFC cells firing rates (Yamashita et al., 2013; Buzsáki and Mizuseki, 2014).

### CA1 and PFC Connectivity

It is well known that the hippocampus closely interacts with a large number of cortex areas, including the PFC, in both monosynaptic and multisynaptic pathways (Griffin, 2015; Ito et al., 2015; Eichenbaum, 2017; Skelin et al., 2018). The ventral hippocampus CA1 region and the proximal subiculum make monosynaptic projections directly to both the excitatory and inhibitory neurons in PFC (Jay and Witter, 1991; Tierney et al., 2004; Hoover and Vertes, 2007). Based on these experimental findings, the pyramidal cells in our CA1 model project to both the pyramidal cells and interneurons in PFC via AMPA synapses. The specifics of the connection are shown in Supplementary Table S6. In the PFC model, 50 pyramidal cells are randomly chosen and divided into five different groups. Each PFC PY cell in one group receives AMPA projections from randomly selected 30 out of 80 PY cells from a specific place cell assembly in CA1. Similarly, the selected CA1 PY cells also form AMPA synapses to all the IN cells in PFC for feed-forward inhibition.

### Spike-Timing-Dependent Plasticity Rule

To investigate the sequence learning capability of the cortex model under CA1 input, we implement STDP for both the PY-PY AMPA synapses and IN-PY GABAa synapses to make sure that the excitation-inhibition balance is maintained throughout the learning process (Vogels et al., 2011). The excitatory STDP for PY-PY AMPA synapses has a classic asymmetric shape (Caporale and Dan, 2008), while the inhibitory STDP for GABAa synapses has a symmetric shape, which has been reported recently in layer V cortical network (D’amour and Froemke, 2015). The formula for the STDP of PY-PY AMPA synapses is shown in Eq. 1, where *W*_*ampa*_ is the current AMPA synaptic strength; *W_*TH*__1_* is the target LTP synaptic strength for AMPA synapses; *p*_1_ is the potentiation factor; d is the depression factor; *τ_*p*__1_* is the LTP time constant; *τ_*d*_* is the LTD time constant. The formula for the STDP of IN-PY GABAa synapses is shown in Eq. 2, where *W*_*gaba*_ is the current GABAa synaptic strength; *W_*TH*__2_* is the target LTP synaptic strength for GABAa synapses; *p*_2_ is the potentiation factor; *τ_*p*__2_* is the LTP time constant. To prevent divergence, the synaptic weights for AMPA and GABAa synapses are restricted in a defined range shown in Eqs 1 and 2. The values of the parameters in the STDP rule is summarized in Supplementary Table S7. Note that our STDP rule assumes a linear integration of the STDP potentiation and depression effect when multiple spikes happen in a short interval. In experimental studies, it has been found that non-linear integration of STDP exists (Wang et al., 2005).

(1) $$\Delta \,W_{\text{LTP}}=\left(W_{T\,H\,1}-W_{a\,m\,p\,a}\right)\cdot p_{1}\cdot e\,x\,p\,\left(-\frac{\Delta \,t}{\tau_{p\,1}}\right)\;i\,f\,\Delta \,t>0$$

$$\Delta \,W_{\text{LTD}}=-\left(W_{T\,H\,1}-W_{a\,m\,p\,a}\right)\cdot d\cdot e\,x\,p\,\left(\frac{\Delta \,t}{\tau_{d}}\right)\;i\,f\,\Delta \,t<0$$

$$W_{a\,m\,p\,a}\in \left[W_{T\,L\,1},W_{T\,H\,1}\right]$$

$$W_{T\,L\,1}=\frac{1}{100}\cdot W_{i\,n\,i\,t_{a\,m\,p}}$$

(2) $$\Delta \,W_{\text{LTP}}=\left(W_{T\,H\,2}-W_{g\,a\,b\,a\,a}\right)\cdot p_{2}\cdot e\,x\,p\,\left(\frac{-\left|\Delta \,t\right|}{\tau_{p\,2}}\right)\;$$

$$W_{g\,a\,b\,a}\in \left[W_{i\,n\,i\,t\,\_\,g\,a\,b\,a\,a},W_{T\,H\,2}\right]$$

### Sequence Replay Analysis

To quantify the sequence replay, similar with previous studies (Ji and Wilson, 2007; Jahnke et al., 2015), we define a matching index (MI) shown in Eq. 3. In this equation, *N* is the total number of neuron pairs from different groups. Since there are five groups in the simulation, each of which have 10 neurons, the value of *N* equals 1000. *n*_*correct*_ is the number of correctly ordered neuron pairs between any two groups and *n*_*wrong*_ is the reversely ordered neuron pairs. When all the neurons fire and form a sequence in the ideal order (group A → group B → group C → group D → group E), the term *n*_*correct*_ will equal to *N* and *n*_*wrong*_ equals to 0. Therefore, the value MI will be 1. For ideal reverse replay in the form (group E → group D → group C → group B → group A), the term *n*_*correct*_ will equal to 0 and *n*_*wrong*_ equals to *N*. Therefore, the value MI will be −1. If the sequence replay is totally random, on average, the term *n*_*correct*_ and *n*_*wrong*_ will be equal. In this case, the value MI will be close to 0.

(3) $$M\,I=\frac{n_{c\,o\,r\,r\,e\,c\,t}-n_{w\,r\,o\,n\,g}}{N}$$

$$N=C\,\left(5,2\right)*n_{w\,i\,t\,h\,i\,n}^{2}=\frac{5!}{2!\,\left(5-2\right)!}*10^{2}=1000$$

### Calculation of Local Field Potential and Background Noise

To compute the local field potential, we sum up the total electric field generated by the transmembrane and postsynaptic currents across all the compartments of the neurons (Nunez and Srinivasan, 2006). The recorded electric potential is computed by Eq. 4 using the source-field model of current monopoles. In the equation, ϕ(*r*,*t*) is the recorded potential at time t and position r. σ is the extracellular conductivity. *N* is the total number of compartments. |r-r_*n*_| is the distance between the compartment n and the recording position.

(4) $$\phi \,\left(\text{r},\text{t}\right)=\frac{1}{4\,\pi \,\sigma }\,\sum_{n=1}^{N}\frac{I_{n}\,\left(t\right)}{\left|r-r_{n}\right|}$$

In our model, the background noise of each neuron is introduced through the noisy AMPA synapses of Poissonian characteristics. In order to quantify the intensity of the background noise, we compute the second moment of the noise current, which is the same as previous studies (Stacey et al., 2009; Canakci et al., 2017).

## Results

### CA1 and PFC Network Activities During Memory Trace Transfer

**FIGURE 1:**
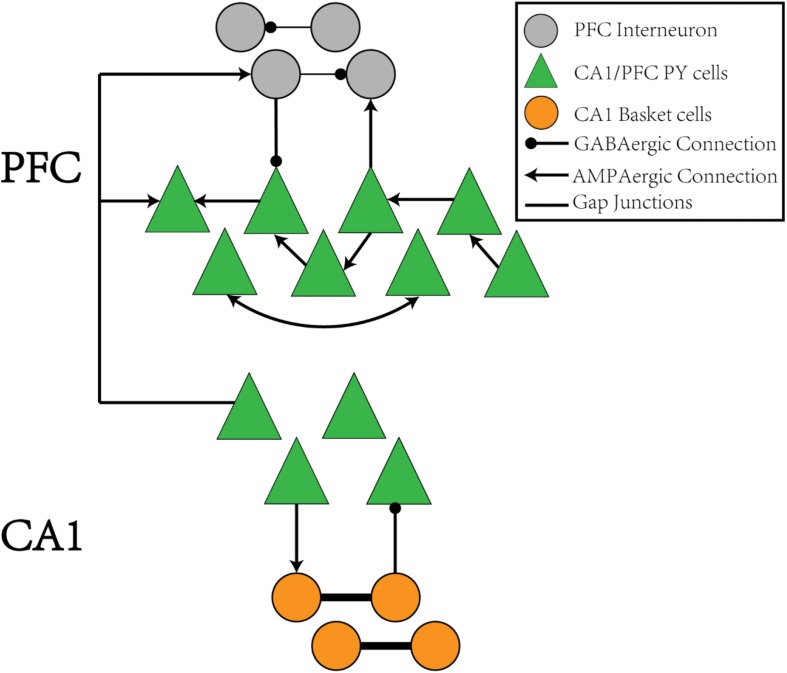
The schematic of the CA1-PFC model. For the CA1 network, the PY cells connects to the IN cells with AMPA synapses, whereas the IN cells project back to the PY cells with GABAa synapses. There is no synaptic connections between the PY cells in CA1. For the PFC network, the PY cells forms unidirectional and bidirectional recurrent connections among each other with a certain probability. The PY cells also project to IN cells with AMPA synapses. The IN cells in PFC have recurrent GABAa connections among each other and project back to the PY cells in PFC through GABAa synapses, Between the CA1 and PFC networks, the PY cells in PFC receive AMPA synaptic inputs from the PY cells in CA1 network. The IN cells in PFC also receive AMPA synaptic inputs from PY cells in CA1.

The CA1/PFC coupling network model is illustrated in the schematic shown in Figure 1. The CA1 network consists of 400 pyramidal (PY) neurons and 100 basket (BS) cells. The PY neurons project to BS cells through AMPA synapses and receive GABA projections from the BS cells. The gap junctions exist between adjacent BS cells. For the PFC network, there are 100 PY neurons and 25 interneurons (IN). The PY neurons form unidirectional and bidirectional AMPA synapses on each other and also send excitatory inputs to the IN neurons. The IN neurons connect back to the PY cells through GABA synapses and also form recurrent inhibitory connections between each other. The details of the network model are explained in the section “Materials and Methods.”

**FIGURE 2:**
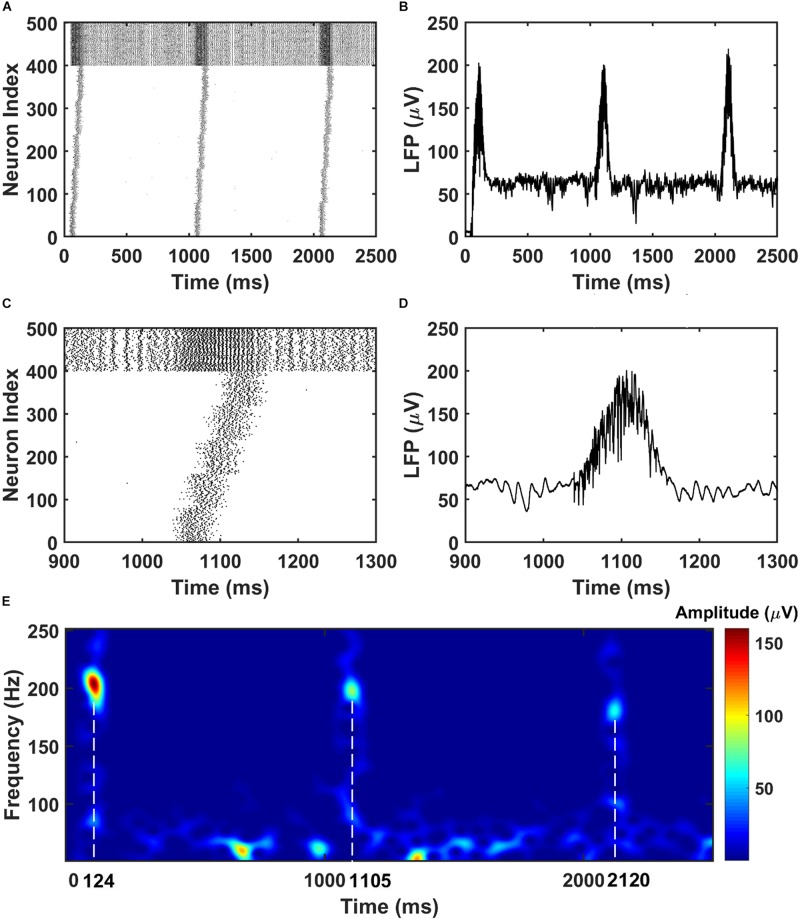
The raster plot and LFP recordings in CA1 network. **(A)** The raster plot of the CA1 network during the simulation (The PY cells are labeled with index 1–400, whereas the BS cells are labeled with index 401–500). Under the sequential noisy inputs, the PY cells in CA1 network shows ordered firing across five different groups. **(B)** The simultaneously recorded LFP shows ripples and sharp waves. **(C)** A zoom-in raster plot of one ripple event in the CA1 network [same index for PY and BS cells as shown in **(A)**]. **(D)** A zoom-in LFP recording during the ripple event shown in **(C)**. **(E)** A spectrogram of the LFP recordings showing ripple range oscillations during sequential replay in the CA1 network.

To mimic the CA3 input that drives the CA1 network, we give sequential noisy inputs (see section “Materials and Methods”) to both the PY neurons and BS neurons in the CA1 network. In this occasion, the PY cells in the CA1 network get depolarized and fire sequentially, as shown in Figures 2A,B. As a consequence, the local field potential (LFP) recordings in the soma layer show ripple transients overlapped on the sharp-waves (Figures 2C,D). Figure 2E is a spectrogram of the LFP recordings in the CA1 network, showing ripple range oscillations around 200 Hz. Also, we examined the timing of the firing activity of PY cells and BS cells in the CA1 network with respect to the phase of the recorded LFP ripples. As shown in Supplementary Figure S1, the result shows obvious phase locking for both PY cells and BS cells, consistent with the previous experimental and modeling literature (Buzsaki et al., 1992; Ramirez-Villegas et al., 2018). Note that, besides the compressed forward sequence replay, by adjusting the input to the CA1 network, our model can also achieve compressed reverse replay and sequential activations in behavioral time scales (Supplementary Figure S2).

**FIGURE 3:**
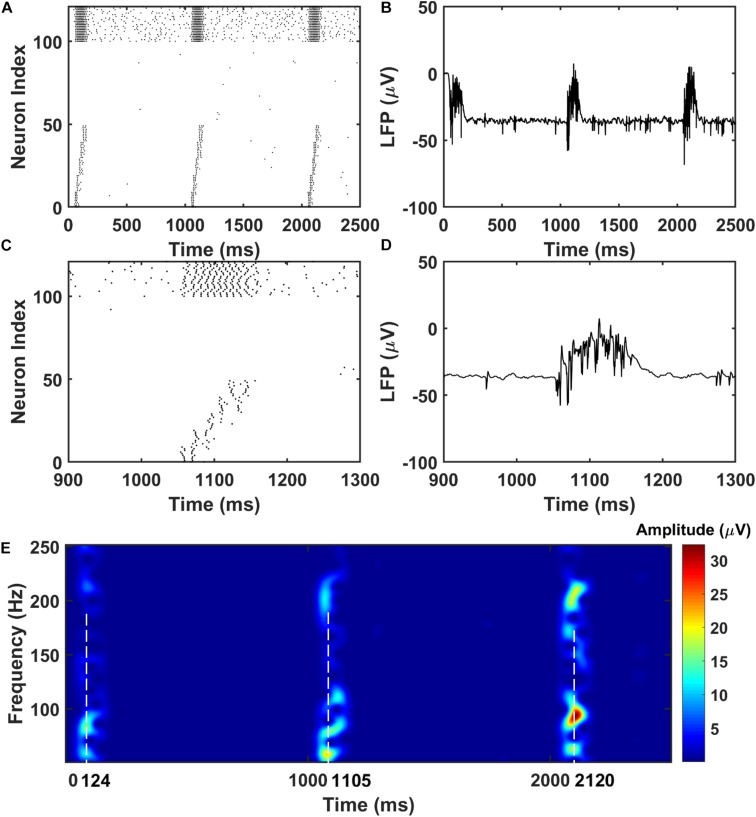
The raster plot and LFP recordings in PFC network. **(A)** The raster plot of the PFC network during the simulation (The PY cells receiving CA1 inputs are labeled as 1–50, whereas the PY cells not receiving CA1 inputs are labeled as 51–100; The IN cells are indexed as 101–125). Under the monosynaptic inputs from CA1, the PY cells in PFC network shows sequential firing. **(B)** The simultaneously recorded LFP in the PFC network. **(C)** A zoom-in raster plot of the PFC network during the ripple event in CA1. **(D)** A zoom-in LFP recording in the PFC network during the sequential firing shown in **(C)**. **(E)** A spectrogram of the LFP recordings showing high gamma and ripple range oscillations during the CA1 ripple events.

For the PFC network, when the CA1 PY cells fire and send excitatory inputs to both the PY cells and IN cells in PFC through monosynaptic connections, the PY cells in PFC fire sequentially (Figures 3A,B). The IN cells also increase their firing frequencies. In the same time, as shown in Figures 3C,D, the LFP recordings in the PFC network exhibit transient oscillations that have components in high gamma (60–100 Hz) and ripple range (100–250 Hz). This can also be seen from the spectrogram of the LFP recordings in PFC network (Figure 3E).

Recent studies on HPC–PFC interactions have revealed various neural activity modulations in both awake and sleep state (Tang et al., 2017; Skelin et al., 2018; Tang and Jadhav, 2019). During sleep, it has been shown that the localized ripple oscillations detected in the LFP recordings at PFC are strongly coupled to the ripple events in the hippocampus (Khodagholy et al., 2017). Also, the coupling strength gets stronger after the animal performs a spatial learning task. Our LFP simulation results are consistent with these observations, suggesting that the ripple-ripple cross-frequency coupling might serve as a communication link between the cortex and hippocampus for memory trace transfer.

### The Sequence Transfer in the Hippocampus-PFC Network

**FIGURE 4:**
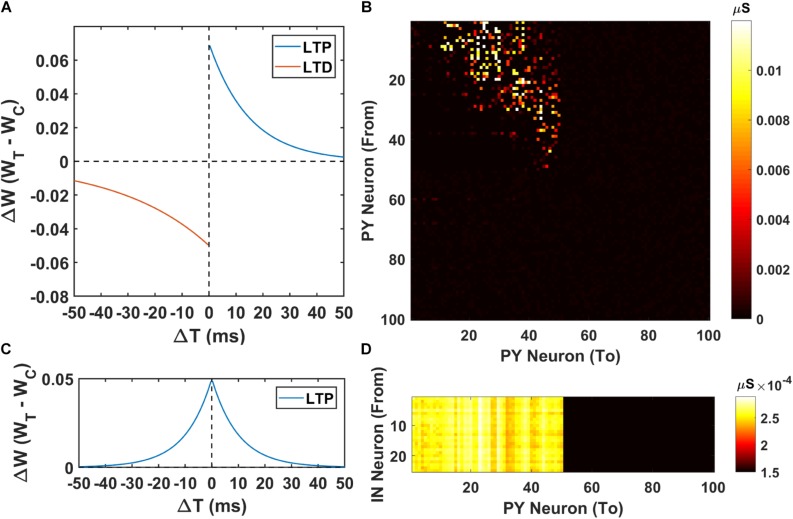
The weight matrices of the PFC network. **(A)** The STDP weight update rule for the PY-PY AMPA synapses in the PFC network. **(B)** The weight matrix of the PY-PY AMPA connections after learning. It can be seen that, for the PY cells that receive CA1 input, the feed-forward connections among them get strengthened, while the feed-backward connections remains weak. **(C)** The STDP weight update rule for the IN-PY GABAa synapses in the PFC network. **(D)** The weight matrix of the IN-PY GABAa connections after learning. Since the first 50 PY cells in PFC network fire sequentially during the learning, all of the corresponding IN-PY connections are strengthened due to the symmetric LTP-only STDP rule for GABAa synapses, whereas the rest connections does not get potentiated much.

Next, we investigate the possibility of the memory transfer through CA1-PFC communication and STDP. The CA1 network generates 5 ripples per second and the simulation time is set to 3 s. The raster plot and LFP recordings in PFC from one representative simulation are shown in Figure 3. When the CA1 network generates ripples, parts of the PY cells in PFC fire sequentially, while all the interneurons (basket cells) in PFC increase their firing rate compared with no-ripple time intervals due to both feed-forward inputs from CA1 PY cells and excitatory inputs from PFC PY cells. Due to the STDP rule, the PY-PY connections and IN-PY connections are updated according to the spike timing of the cells (Figures 4A,C). As shown in Figure 4B, by the end of one representative simulation (3000 ms training time), the feed-forward AMPA connections among the PY cells that receive direct CA1 inputs are strengthened, whereas the feed-backward AMPA connections are weakened. Since the inhibitory STDP rule is symmetric and LTP-only, all the IN-PY GABAa connections for the PY cells that receive direct CA1 inputs are stronger by the end of the simulation (Figure 4D). However, for the PY cells that do not directly receive CA1 input, the strengths of their GABAa synapses are almost unchanged because of their sparse firing activities. Therefore, under the STDP rule for both excitatory and inhibitory synapses in the PFC network, the sequence initiated in CA1 is successfully transferred to the PFC network and stored in the recurrent synaptic connections of the cortical cell populations.

### The Sequence Replay in PFC Network Induced by Cell-Specific Input

After the successful storage of the CA1 sequence, we investigate the retrieval of the memory in the PFC network by cell-specific stimulations to part of the PY cells in PFC network. The cell-specific input induced replay represents the scenarios where the memory is recalled by the sensory stimulation or natural cues, which is commonly observed in studies on contextual fear conditioning (Maren et al., 2013).

**FIGURE 5:**
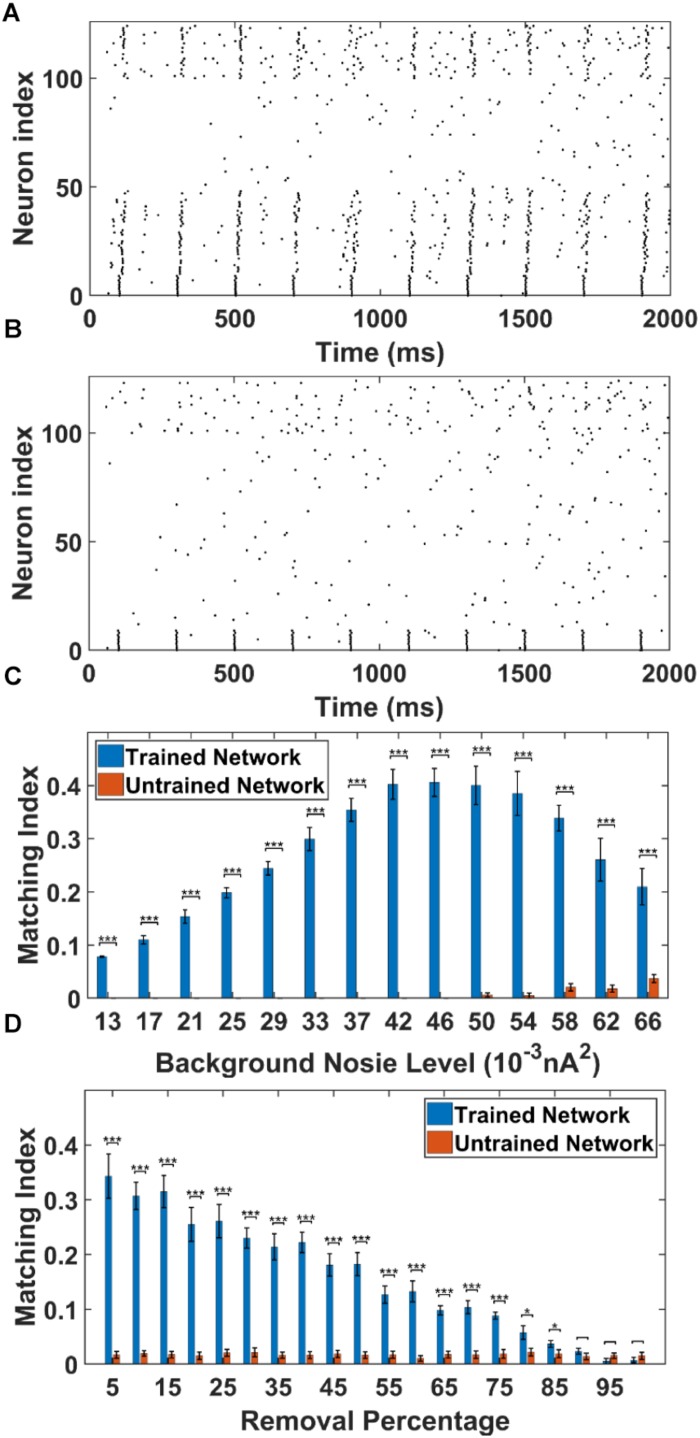
The sequence replay in PFC network induced by cell-specific current injection. **(A)** When the first 10 PY cells in the trained PFC network are given a short 5 ms current injection, the downstream PY cells are recruited by the feed-forward AMPA projections and a sequence is induced (noise level = 0.053 nA2). All the neurons are indexed the same as in Figure 3A. **(B)** For the untrained PFC network, the PY cells exhibit random firing and the activation of first 10 PY cells do not induce sequence replay. All the neurons are indexed the same as in Figure 3A. **(C)** The sequence matching index MI induced by strong cell-specific input depends on background noisy input to the PY cells. Note that the MIs of sequence replay in trained network are significantly different from those in the untrained network for all the noise input levels (^∗∗∗^ significant at *p* < 0.001, rank sum test). **(D)** The sequence replay MIs depend on the PY-PY connection integrity. Massive loss of AMPA connections between PY cells will result in insufficient EPSPs to depotentiate the membrane potentials of downstream PY cells, which will in turn suppress the sequence replay. However, the replay MI is relatively robust to connection loss for up to 75% connection loss (^∗^ significant at *p* < 0.05, ^∗∗∗^ significant at *p* < 0.001, rank sum test).

To apply the cell-specific input, we inject a train of 5 Hz 5 ms duration 0.7 nA step currents into the soma of the first 10 PY cells in the trained PFC network and compute the MI of sequence replay (noise level = 0.053 nA^2^, see section “Materials and Methods”). The MI value quantitatively measures the replay quality. A perfect forward replay will have a MI value of 1, whereas the random replay will have a MI value of 0. We also perform simulations in the untrained PFC network using the same stimulation protocol as control group. The raster plot of the trained PFC network is shown in Figure 5A. It can be seen that, under the current injection, the first 10 PY cells fire and quickly recruit the rest of the downstream PY cells to form a sequence in a temporally compressed manner (∼30 ms). This replay mechanism relies on the strengthened AMPA synapses between PY cells and a sufficient number of recurrent PY-PY AMPA connections. The firing of each PY cell will elicit an excitatory post-synaptic potentials (EPSPs) in the downstream PY cells, which elevates the membrane potential. If the postsynaptic PY cell receives multiple EPSPs from multiple presynaptic PY cells in a short time interval, it is more likely to fire. For the untrained network case, the raster plot is shown in Figure 5B. It can be seen that even though the first 10 PY cells fire under the external stimulation, the rest of the PY cells do not fire to form a sequence. This is due to the weak PY-PY AMPA connections in the untrained network such that the firing of first 10 PY cells cannot generate large enough EPSPs in the downstream PY cells. To further quantitatively explore the possible factors that can affect the sequence replay, we examine two parameters that can change the sequence MI in PFC network: the AMPA noisy input level and the recurrent AMPA connection degradation.

As shown in Figure 5C, we plot and compare the stimulation-induced sequence MI for the trained network and untrained network under different background noise levels. For each replay event, a 50 ms time window is applied to calculate the MI value. Note that, the MIs of the sequence replay in the trained PFC network differ significantly from their counterparts in the untrained PFC network at all the noise levels (*p*-value < 0.001, rank sum test). In the trained network, the sequence MI reaches maximum at intermediate noise level. Weak and strong noise levels will both degrade the MI in different ways. In the case of weak noisy input, the membrane potentials of the PY cells are far below the voltage threshold. The PY cells are less likely to fire, which in turn decreases the number of PY cells recruited during the replay. On the other hand, under the strong noisy input, the membrane potentials of PY cells are driven closer to the voltage threshold. This effectively increases the randomness of firing for PY cells, which degrades the ordered firing and lower the MI value. Therefore, the intermediate background noise level balances the tradeoff between sufficiently large membrane potentials and the randomness of firing, which leads to the most ordered replay and the highest MI value. In the untrained network, for the low noise level cases, the firings of the first 10 PY cells are not sufficient to induce firing in the downstream PY cells, which leads to MI value of 0. However, as the noise level increases, the membrane potentials of the downstream PY cells are depolarized so that the firing of the first 10 PY cells will drive part of the PY cells to fire. Therefore, the MI starts to increase. These results indicate that a proper spontaneous background noise level will affect the sequence replay and is very important in the memory retrieval process.

In order to examine the effect of AMPA connection loss on the sequence replay, we eliminate a random number of AMPA connections in both the trained and untrained PFC network by setting the conductance to 0 and compute the MI value under the same stimulation protocol as before. The resulting sequence MIs under different proportion of AMPA connection loss is shown in Figure 5D. In the trained network, as more and more AMPA connections are eliminated, the MI value gets lower because of less PY cell firings due to the decreasing number of EPSPs from the presynaptic PY cells. On the other hand, the sequence MI is actually robust to recurrent AMPA connection loss. Even though the MI value of sequence replay drops with an increasing loss of AMPA connections, the MI value for the trained network is still significantly different from that of the untrained network for up to 75% AMPA connection loss (*p*-value < 0.001, rank sum test). These results indicate that under abundant recurrent connections, the trained network is relatively robust to connectivity damage or degradation in order to successfully recall the memory.

### The Sequence Replay in PFC Network Induced by Non-specific Background Noise

Besides the stimulus-induced sequence replay discussed above, we also investigate the spontaneous replay induced by non-specific background noises to all the PY cells in both the trained and untrained network. In other words, we want to examine the possibility of emerging memory retrieval without specific external cues. The non-specific spontaneous noise induced replay resembles the experimentally observed spontaneous reactivation events in the neocortex, where no specific inputs are present (Kenet et al., 2003; Luczak et al., 2007; Bermudez Contreras et al., 2013). The generation and control of these spontaneously emerging replays could be attributed to the neuromodulator system, which exerts a widespread modulation of the overall excitability in a large neuronal network (Hasselmo et al., 1995; Hasselmo, 1999).

**FIGURE 6:**
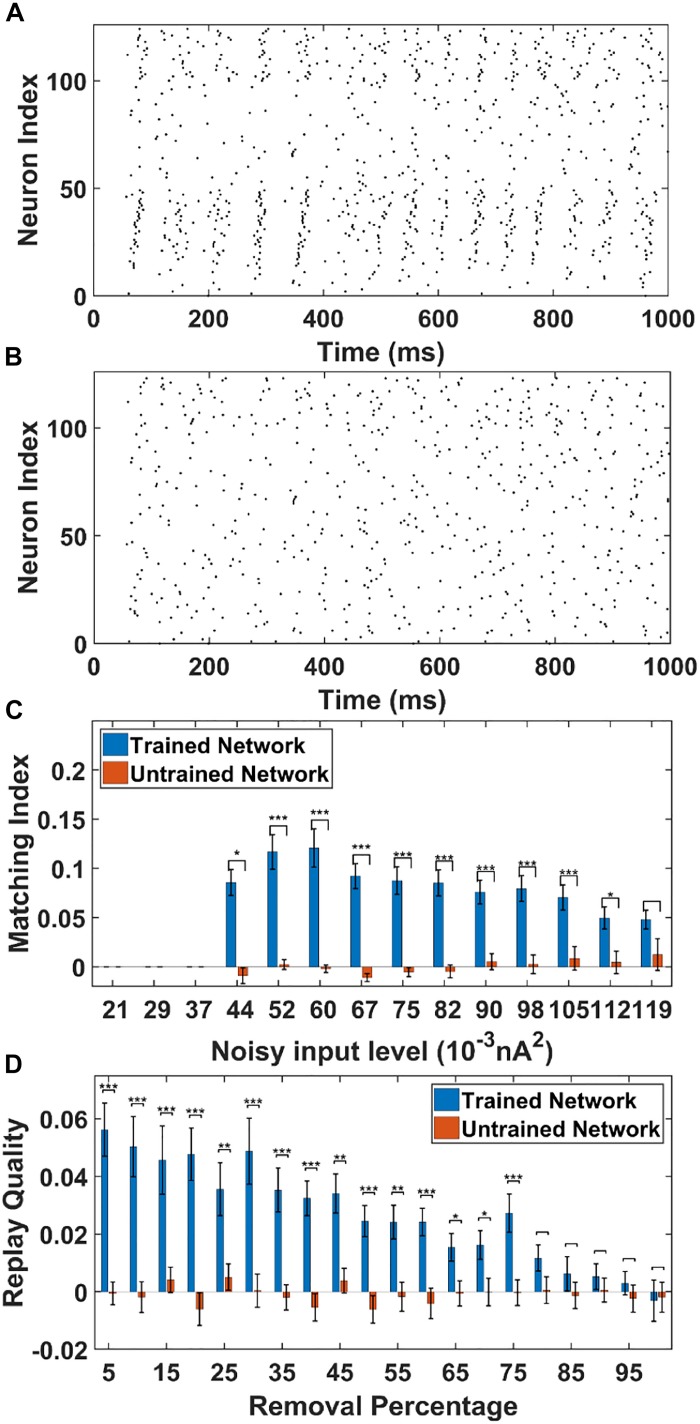
The sequence replay in PFC network induced by non-specific spontaneous noisy input. **(A)** Under the noisy input to the trained PFC network, the sequence emerges randomly among the PY cell populations with a varying replay length and initiating neurons (noise level = 0.052 nA2). All the neurons are indexed the same as in Figure 3A. **(B)** For the untrained PFC network, the firing activity is random and no sequence replay emerges. All the neurons are indexed the same as in Figure 3A. **(C)** The MIs of sequence replay in the trained network for different noisy input levels. For low noisy inputs, sequence replay cannot emerge. The MI value reaches the maximum for intermediate noisy input levels. For high noise level, the sequence replay in the trained network do not differ significantly from that in the untrained one (^∗^ significant at *p* < 0.05, ^∗∗∗^ significant at *p* < 0.001, rank sum test). **(D)** The MIs of sequence replay in the trained network for different AMPA synapse losses. Even though the MIs are low, they still differ significantly from those in the untrained PFC network (^∗^ significant at *p* < 0.05, ^∗∗^ significant at *p* < 0.01, and ^∗∗∗^ significant at *p* < 0.001, rank sum test).

To simulate this, we adjust the background noise parameters to the PY cells. In this case, the PY cells in the PFC network do not receive any cell-specific stimulation. Each PY cell in the PFC network receives statistically the same background noise. The raster plot of the trained network from one representative simulation (noise level = 0.052 nA^2^) is shown in Figure 6A. The raster plot of the untrained network under the same simulation condition is shown in Figure 6B. It can be seen that under the non-specific spontaneous noisy input, the sequence replay can indeed emerge randomly for the trained network. However, compared with the sequence replay induced by cell-specific current injection, the sequence length and the initiating neurons for each recall can vary. For the untrained network, the noise induced sequence replay is not possible. Similar with the cell-specific stimulation case, we quantitatively investigate two parameters that might affect the MI under spontaneous noisy input, namely the AMPA noisy input level and the AMPA connection loss.

As shown in Figure 6C, the sequence MIs are compared between the trained and untrained PFC network for different noisy input levels. For the trained PFC network at low noisy input level, the MI is very small. As the noise level increases, sequence replay starts to emerge and the MI reaches the maximum value and then drops for larger noise levels. This is because when the noise level is low, the membrane potential of the PY cells are far below the voltage thresholds. Therefore, the firing of PY cells is relatively sparse and an ordered replay that recruits a large number of PY cells is hard to achieve, leading to a low MI. When the noise increases to an optimal intermediate value, the membrane potential is elevated to a sufficient level and the firing of certain PY cells is enough to induce the firing of downstream PY cells. If the noise further increase to an overly high level, the randomness firing overwhelms the network and the MI degrades. For the untrained network, the MI values remain low for all the noisy input levels. Note that, even though the MI values for the trained network under spontaneous noisy input are smaller than that for the previously discussed cell-specific input case, the MIs are still significantly different from those for untrained network (*p* < 0.001, one-sided rank sum test). Here, by using one-sided rank sum test, we are only treating the forward replay (MI close to + 1) as significant events. These results show that given a proper noise level, the memory replay is indeed possible under non-specific background noise in the trained PFC network.

For the AMPA connection degradation condition, similar to the cell-specific case, we randomly delete the same AMPA synapses between PY cells in both the trained and untrained network. Then, spontaneous noisy inputs are given to all the PY cells in the PFC network and sequence MIs are computed. As shown in Figure 6D, for the trained network, the MI drops as the number of removed AMPA connections increases, since the total EPSPs from presynaptic PY cells to one postsynaptic PY cell are decreasing with more AMPA synapse losses. However, for the untrained network, the MI values do not change much and remain low for different AMPA connection losses. Still, the network is relatively robust to connection loss, meaning that the MI values for trained network are significantly different from those in the untrained network for up to 75% AMPA connection loss.

### The Effect of SWR Number and Background Noise on Memory Retrieval

It has been reported that during the early stage of PFC engram cell formation, the natural cues cannot induce the memory recall. However, the engram cells can be activated by optogenetic stimulations, which will lead to successful memory retrieval (Kitamura et al., 2017; Roy et al., 2017). Also, the spine density of PFC engram cells in the later days of learning is significantly higher than the early days (Kitamura et al., 2017). These findings lead to the postulation of “silent engrams” and “active engrams,” which refer to the neurons that encode memory, having weak or strong synaptic connections between them, respectively. To explore this idea from a computational perspective, we simulate the network for two different numbers of SWRs during training (2000 ms, 10 SWRs and 3800 ms, 19 SWRs) and test the sequence replay using strong current injection to the first 10 PY cells.

**FIGURE 7:**
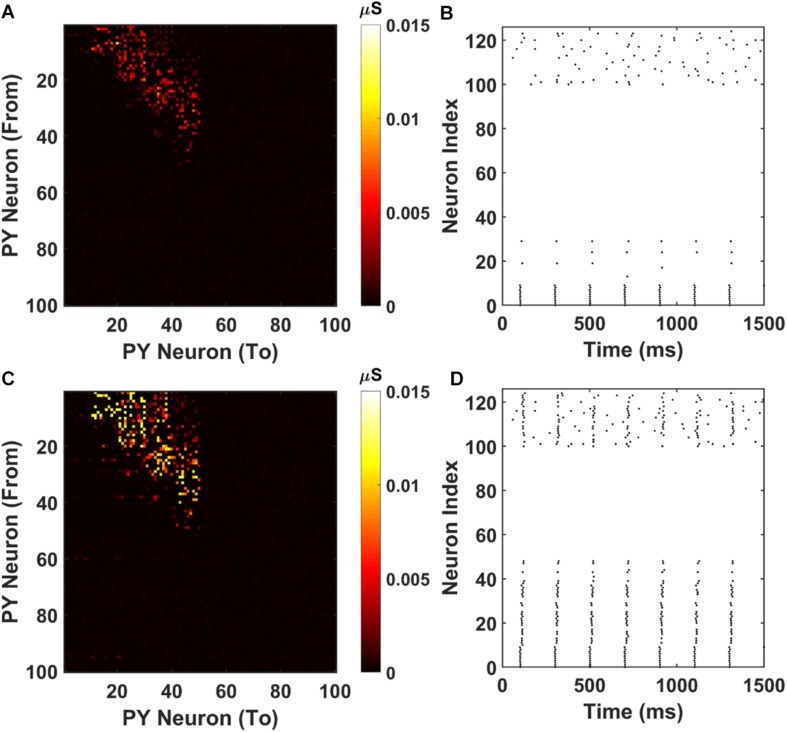
Weight matrices and raster plots for silent engram assembly and active engram assembly under cell-specific strong input. **(A)** The recurrent AMPA connection matrix between PY cells in the PFC network after short training (2000 ms). The feed-forward connections are strengthened, but not strong. **(B)** The raster plot of the PFC network under cell specific current injection. The firing of the first 10 PY cells are not sufficient to induce massive firing of the downstream PY cells. All the neurons are indexed the same as in Figure 3A. **(C)** The recurrent AMPA connection matrix between PY cells in the PFC network after long training (3800 ms). The feed-forward connections are stronger compared with **(A)**. **(D)** The raster plot of the PFC network under same input as **(B)**. The firing of the first 10 PY successfully induce a sequence replay in the downstream PY cells. All the neurons are indexed the same as in Figure 3A.

The resulting AMPA recurrent connection matrix and the sequence replay are shown in Figure 7. It can be seen that for the shorter training time (2000 ms), the feed-forward connections in the PFC network are already established through LTP (Figure 7A). However, under the stimulation to the first 10 PY cells, the sequence cannot be retrieved because of the weak AMPA connections that are not sufficient to depolarize the membrane potential (Figure 7B). For the longer training time, the AMPA connections among the PY cells in PFC are further potentiated (Figure 7C) and the sequence can be replayed by the same stimulation on the first 10 PY cells (Figure 7D). These results support the idea that the “silent engrams” can get mature with longer training and eventually turn into “active engrams” for successful memory retrieval.

**FIGURE 8:**
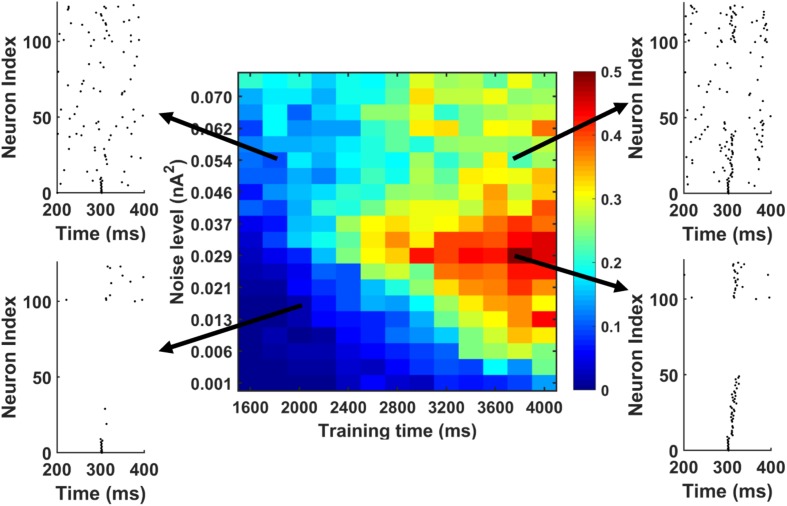
The MI as a function of training time and noise level during memory replay. The first 10 PY cells get strong current injection to initiate the replay. In general, under fixed background noise level, the longer training time is, the better the replay will be. Under fixed training time, moving from low noise to intermediate noise level improves the sequence replay. However, further increasing the noise level will degrade the MI. Also, by increasing the noise level from low to intermediate level, the same sequence MI can be achieved using shorter training time (see the solid black arrow).

Further, we investigated the possibility whether we could actually achieve successful memory recall among the “silent engrams.” To simulate this, during memory recall, we apply different background noise levels to the PY cells in the PFC networks going through different training times. The result is shown in Figure 8. The red color indicates successful sequence replay with high MI values, whereas the blue color stands for poor sequence replay with low MI values. It can be seen that indeed, for different noise levels, longer training time will typically result in better sequence replays. Also, by increasing the background noise levels during memory retrieval, good sequence replay can be achieved even for shorter training time. The elevated background noise level effectively increases the average membrane potential and hence the excitability of PY cells. This can be achieved either naturally through neuromodulator system (Hasselmo, 1999; Constantinople and Bruno, 2011) or manually through transcranial electrical stimulation (Bikson et al., 2004; Vöröslakos et al., 2018). However, the boosting effect of the noisy inputs does not extend for high noise levels. Further increasing the noise level will hurt the sequence replay and result in low MI values due to excessive firing dominated by randomness.

## Discussion

In this work, we build a biophysical model that includes both the hippocampus CA1 network and the PFC network to study the memory transfer and reactivation. Under sequential input, the pyramidal cells in the CA1 network exhibit both ordered replay activities and ripples in the LFP recordings, consistent with the experimental findings (Diba and Buzsáki, 2007). Also, as shown in Supplementary Figure S1, the pyramidal cells and the basket cells fire at preferred ripple phases, consistent with previous experimental study (Buzsaki et al., 1992). Besides producing the electrophysiological signals that are consistent with the existing literature, our model also makes a few assumptions and predictions that can be tested in future experimental studies. First, our model suggests the existence of cortical ripples in PFC network coupled to the hippocampal ripples. This is already confirmed with the recent study suggesting mPFC-hippocampal LFP coupling during NREM sleep. However, it is still unknown if this cortical-hippocampal ripple coupling happens during awake state too. Second, our model indicates that the sequence reactivation in PFC network can be achieved through both cell-specific stimulation and spontaneous background synaptic noise. To test the former case, the optogenetics can be used to selectively stimulate the neurons that participate in specific sequence reactivation and examine the animal behavior in memory tasks, similar to the fear conditioning experiments. To test the latter case, one can quantitatively control the concentration of neuromodulators and investigate whether the background noise level change has a correlation with the performance of the animal in memory tasks. Third, our model predicts that the transferred memory in the PFC network is relatively robust to the recurrent connection loss between the pyramidal cells. The memory can be successfully retrieved under sufficient inputs and background noise level. In a recent relevant computational study (Acker et al., 2019), it was demonstrated that the Hebbian plasticity of synaptic strength can help preserve the place fields of pyramidal cells in hippocampus CA1 regions and the orientation tuning curve in neurons from visual cortex even under random input synaptic turnovers. Similarly, in another study of memory retention under synapse turnover (Fauth and van Rossum, 2019), the author showed that by performing self-reactivation during rest time, the synaptic strength can be reinforced to maintain the cell assembly against the connection loss. When comparing our work to these two studies, even though the type of synapse loss is different, the successful recovery of activation pattern all depends on the sufficient inputs that are compensated by two mechanisms separately (one is through the background noise level, the other is Hebbian plasticity and reweighting of synapses). Finally, our modeling result suggests that an optimal background noise level exists for the memory reactivation in both the “well-trained” and “less-trained” networks. Similar to the second prediction, this can be tested by adjusting the neuromodulator level or using electrical stimulation to regulate the excitability of the neurons participating the sequence reactivation.

Many modeling studies have been conducted to model the CA1 and CA3 networks in the hippocampus. Among these works, most of them are focusing on the mechanism of ripple generation and the sequence storage and reactivation. Under external excitatory inputs from CA3 or entorhinal cortex, the CA1 ripples have been proposed to be generated either by pyramidal to pyramidal coupling through gap junctions (Traub and Bibbig, 2000; Traub et al., 2012), or by the pacing effect of feedback inhibition to the pyramidal cells from interneurons (Taxidis et al., 2012; Cutsuridis and Taxidis, 2013; Malerba et al., 2016; Ramirez-Villegas et al., 2018). Our model falls within the second category in that the pyramidal cell firing are paced by the synchronous firing of interneurons and no gap junctions exist between pyramidal cells. In terms of the generation mechanism of sequence replay in CA1 network, since the CA1 region lacks recurrent pyramidal-to-pyramidal connectivity, most modeling works suggest that the replay results from the sequential input from the CA3 network (Cutsuridis and Hasselmo, 2011; Taxidis et al., 2015; Malerba et al., 2016). Recently, it has been suggested that by modifying the synaptic strength from CA3 to CA1 and the feedback inhibition between interneurons and pyramidal neurons in CA1, the network can exhibit sequence replay (Ramirez-Villegas et al., 2018). The sequence replay generation mechanism in our modeling work is similar to the first group. Instead of explicitly modeling the CA3 network, we give sequential excitatory input to both the pyramidal cells and the basket cells in the CA1 network to induce sequence replay. Compared to the above modeling works, our model focuses more on the memory trace transfer from CA1 to PFC network and the different mechanisms and conditions affecting the reactivation of the transferred memory traces.

It should be noted that our model mainly focuses on the memory trace transfer from CA1 to PFC via monosynaptic connections. This model structure is a simplified version that covers part of the HPC-PFC networks. As mentioned before in the section “Materials and Methods,” anatomical studies show that besides the direct monosynaptic connection from ventral CA1 to PFC, there are also abundant indirect multisynaptic pathways from CA1 to PFC via thalamus and connections from CA3 to PFC via lateral entorhinal cortex. A more comprehensive model including intermediate brain networks can be built to further investigate the memory transfer between hippocampus and PFC in multiple time scales ranging from seconds to hours and days. Also, it should be noted that our model does not intend to reproduce all the physiological phenomena during memory transfer and consolidation observed in animal experiments. For example, after the forward sequence transfer, our model does not generate bi-directional sequence replays in the PFC network. Also, in this study, we do not model the signature LFPs (cortical slow oscillations and spindles) that are important for memory consolidation during slow-wave sleep. Finally, in the current paper, the memory trace transfer is demonstrated as an equivalent transfer of sequential activity from hippocampus to the PFC. We want to clarify that the general memory consolidation and transfer is much more complicated and involves the integration of new memory with related existing hippocampal-dependent memories in the distributed cortical networks.

## Data Availability Statement

The datasets generated for this study are available on request to the corresponding author.

## Author Contributions

XL and DK conceived the research idea and wrote the manuscript. XL wrote the code for the computational models, analyzed the simulation data, and prepared the figures.

## Conflict of Interest

The authors declare that the research was conducted in the absence of any commercial or financial relationships that could be construed as a potential conflict of interest.
