# Supplementary material for: Hippocampal-Cortical Memory Trace Transfer and Reactivation Through Cell-Specific Stimulus and Spontaneous Background Noise
Source: Front Comput Neurosci. 2019 Sep 24;13:67. doi: 10.3389/fncom.2019.00067 (PMC6798041; doi:10.3389/fncom.2019.00067)

Supplementary Material

# Supplementary Tables

**Supplementary Table 1. CA1 neuron model.**

| Neuron Type | Compartment | Diameter/um | Length/um |
| --- | --- | --- | --- |
| Pyramidal | Soma | 20 | 20 |
|  | Basal dendrite | 2 | 200 |
|  | Apical dendrite 1 | 2 | 150 |
|  | Apical dendrite 2 | 2 | 150 |
|  | Apical dendrite 3s | 2 | 150 |
| Basket | Soma 1 | 3.183 | 3.33 |
|  | Soma 2 | 3.183 | 3.33 |
|  | Soma 3 | 3.183 | 3.33 |

**Supplementary Table 2. CA1 network connections.**

| Pre | Post | Type | Location | Connectivity | $\boldsymbol{\tau}_{\mathbf{1}}$/ms | $\boldsymbol{\tau}_{\mathbf{2}}$/ms | Weight/nS | Delay/ms |
| --- | --- | --- | --- | --- | --- | --- | --- | --- |
| PY | BS | AMPA | soma | Each BS cell receives input from nearest 50 PY cells | 0.2 | 1.0 | 0.016 | 1.5 |
| BS | PY | GABAa | soma | Each PY cell receives input from nearest 20 BS cells | 1.5 | 8.0 | 0.275 | 1.5 |
| BS | BS | Gap | soma | Each BS cell forms gap junctions with nearest 4 BS cells | NA | NA | 0.1 | 0.1 |

**Supplementary Table 3. Parameters of the input to CA1 network**

| Duration/ms | Number of input pulses | Start time shift/ms | Total duration |
| --- | --- | --- | --- |
| 35 | 5 | 15 | 95 |

**Supplementary Table 4. PFC pyramidal cell and interneuron physical properties.**

| Neuron Type | Compartment | Diameter/$\boldsymbol{\mu m}$ | Length/$\boldsymbol{\mu m}$ |
| --- | --- | --- | --- |
| PY | Soma | 10.14 | 75.00 |
|  | Axon | 1.10 | 113.22 |
|  | Basal dendrite | 1.00 | 150.00 |
|  | Apical dendrite 1 | 3.40 | 400.00 |
|  | Apical dendrite 2 | 2.60 | 400.00 |
| IN | Soma1 | 42 | 53 |
|  | Axon | 0.7 | 113.22 |

**Supplementary Table 5. PFC network connections.**

| Pre | Post | Type | Location | Connectivity | $\boldsymbol{\tau}_{\mathbf{1}}$/ms | $\boldsymbol{\tau}_{\mathbf{2}}$/ms | Weight/nS | Delay/ms | | |
| --- | --- | --- | --- | --- | --- | --- | --- | --- | --- | --- |
| PY | PY | AMPA | Bdend | Bidirectional(P=0.12)  Unidirectional(P=0.25) | 0.6 | 4.3 | 0.3 | 4±0.9 | | |
| PY | IN | AMPA | Soma | Each IN cell receives inputs from 10 randomly chosen PY cells. | 0.3 | 5.5 | 0.4 | 0.6±0.2 |  |  |
| IN | PY | GABAa | Soma | Each IN connects to all the PY cells | 1.5 | 14 | 0.15 | 1.8±0.8 | |  |
| IN | IN | GABAa | Soma | Each IN cell connects to 10 randomly chosen IN cells. | 3 | 24 | 1 | 1.8±0.8 | |  |

**Supplementary Table 6. CA1-PFC interconnections.**

| Pre | Post | Type | Location | Connectivity | $\boldsymbol{\tau}_{\mathbf{1}}$/ms | $\boldsymbol{\tau}_{\mathbf{2}}$/ms | | Weight/nS | Delay/ms |
| --- | --- | --- | --- | --- | --- | --- | --- | --- | --- |
| CA1 PY | PFC PY | AMPA | Adend1 | Each PFC PY receives randomly chosen 30 CA1 PY inputs in one group | 0.6 | | 4.3 | 0.8 | 5±1.5 |
| CA1 PY | PFC IN | AMPA | soma | Each PFC IN receives inputs from all the CA1 PY neurons | 0.6 | | 4.3 | 0.25 | 5±1.5 |

**Supplementary Table 7. Values for STDP rule parameters.**

| PY-PY STDP | | | | | IN-PY STDP | | |
| --- | --- | --- | --- | --- | --- | --- | --- |
| p1 | d | $\tau_{p1}$/ms | $\tau_{d}$/ms | $W_{TH1}$/nS | p2 | $\tau_{p2}$/ms | $W_{TH1}$/nS |
| 0.07 | 0.05 | 15 | 34 | 12 | 0.05 | 10 | 0.75 |

**Supplementary Figure 1.**

(a) The histogram of the CA1 PY cell firing relative to the phase of the ripples. (b) The histogram of the CA1 BS cell firing relative to the phase of the ripples.


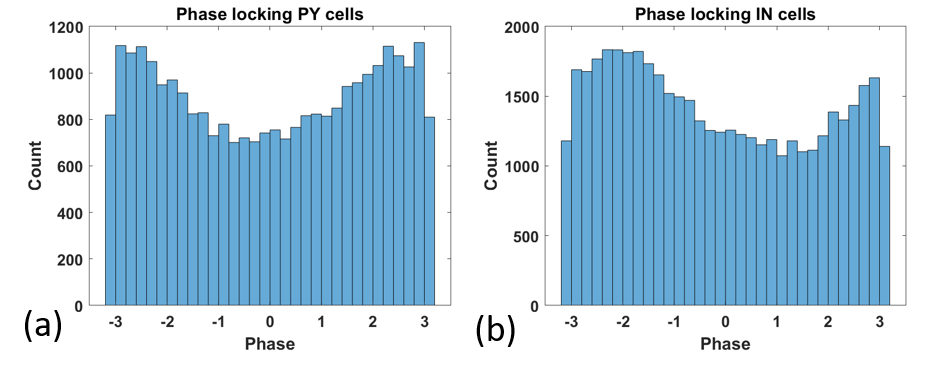


**Supplementary Figure 2.**

(a) The raster plot of CA1 PY cells and BS cells under sequential inputs that are in second-scale, similar to behavioral states. (b) The raster plot of CA1 PY cells and BS cells under reversed sequence replay under the reversed sequential input that are in millisecond-scale.


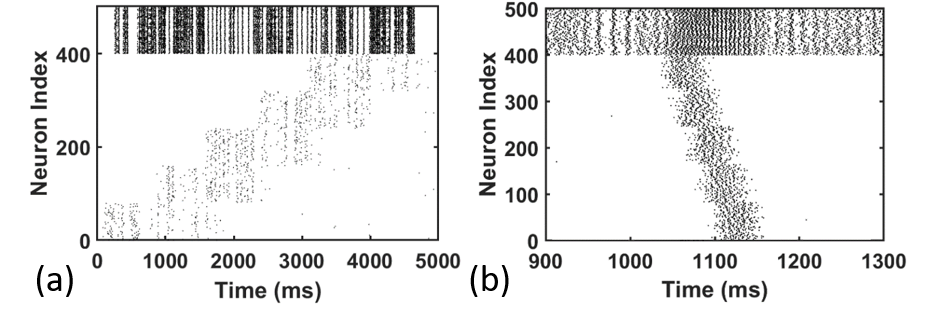

Supplement: Supplementary file 1 [file Table_1.docx]
